# Comparison of echocardiographic methods for calculating left ventricular mass in elite rugby football league athletes and the impact on chamber geometry

**DOI:** 10.3389/fspor.2023.1270444

**Published:** 2023-09-13

**Authors:** Rebecca McGregor-Cheers, Lynsey Forsythe, Robert Cooper, Christopher Johnson, Nicholas Sculthorpe, Michael Papadakis, Nathan Mill, Matt Daniels, Geert Kleinnibbelink, Keith George, David Oxborough

**Affiliations:** ^1^ Research Institute for Sports and Exercise Science, Liverpool John Moores University, Liverpool, United Kingdom; ^2^ Cardiology Department, University Hospitals Bristol and Weston NHS Foundation Trust, Bristol, United Kingdom; ^3^ Sport and Physical Activity Institute, University of the West of Scotland, Glasgow, United Kingdom; ^4^ Cardiovascular Sciences Research Centre, St Georges University of London, London, United Kingdom; ^5^ St Helens Rugby Football League Club, St Helens, United Kingdom; ^6^ Department of Cardiology, Research Institute for Health Sciences, Radboud University Medical Center, Nijmegen, Netherlands

**Keywords:** athletes heart, echocardiography, left ventricle, left ventricular mass, rugby, left ventricular geometry

## Abstract

**Background:**

Recommendations for the echocardiographic assessment of left ventricular (LV) mass in the athlete suggest the use of the linear method using a two-tiered classification system (2TC). The aims of this study were to compare the linear method and the area-length (A-L) method for LV mass in elite rugby football league (RFL) athletes and to establish how any differences impact the classification of LV geometry using 2TC and four-tier (4TC) classification systems.

**Methods:**

Two hundred and twenty (220) male RFL athletes aged 25 ± 5 (14–34 years) were recruited. All athletes underwent echocardiography and LV mass was calculated by the American Society of Echocardiography (ASE) corrected Linear equation (2D) and the A-L method. Left ventricular mass Index (LVMi) was used with relative wall thickness to determine geometry in the 2TC and with concentricity and LV end diastolic volume index for the 4TC. Method specific recommended cut-offs were utilised.

**Results:**

Higher values of absolute (197 ± 34 vs. 181 ± 34 g; *p* < 0.0001) and indexed (92 ± 13 vs. 85 ± 13 g/m^2^; *p* < 0.0001) measures of LV mass were obtained from A-L compared to the linear method. Normal LV geometry was demonstrated in 98.2% and 80% of athletes whilst eccentric hypertrophy in 1.4% and 19.5% for linear and A-L respectively. Both methods provided 0.5% as having concentric remodelling and 0% as having concentric hypertrophy. Allocation to the 4TC resulted in 97% and 80% with normal geometry, 0% and 8.6% with eccentric dilated hypertrophy, 0% and 7.7% with eccentric non-dilated hypertrophy, 1.4% and 0.5% with concentric remodelling and 1.4% and 3% with concentric non-dilated hypertrophy for linear and A-L methods respectively. No participants had concentric dilated hypertrophy from either methods.

**Conclusion:**

The linear and A-L method for calculation of LV mass in RFL athletes are not interchangeable with significantly higher values obtained using A-L method impacting on geometry classification. More athletes present with eccentric hypertrophy using 2TC and eccentric dilated/non-dilated using 4TC. Further studies should be aimed at establishing the association of A-L methods of LV mass and application of the 4TC to the multi-factorial demographics of the athlete.

## Introduction

The athlete's heart refers to physiological adaptation derived from repetitive alterations in cardiac work encountered during long-term exercise training (1). These adaptations are heterogeneous with the magnitude and type of change dependent on many factors including age, gender, body size, sporting discipline and training status (2). The impact on left ventricular (LV) geometry includes increases in cavity dimension, wall thickness and overall LV mass (3–5). Although rare, potential pathological forms of hypertrophy such as hypertrophic cardiomyopathy can create a diagnostic dilemma with athlete's heart causing similar changes in LV geometry (6).

Echocardiography allows the assessment of LV geometry (7) and has traditionally been classified in accordance with the 2-tiered classification (2TC) utilising a combination of scaled LV mass to body surface area [LV Mass Index (LVMi)] and relative wall thickness (RWT) (7, 8). More recently, a 4-tiered classification (4TC) of LV geometry has been developed, distinguishing geometry via the absence or presence of increased LV concentricity^2/3^ [LV mass/end-diastolic volume (EDV)^2/3^], LVMi and LV dilation (LVEDV/BSA) (9). When compared to the 2TC system, the 4TC system has demonstrated better risk-stratification for adverse cardiovascular events in hypertensive patients and the general population (10, 11). Studies in athletes have suggested that further categorisation by the 4TC may be beneficial to more accurately articulate geometrical adaptation (12, 13).

LV mass is a strong predictor of cardiovascular events (14, 15) with its assessment being key to understanding and defining LV geometry. Numerous methods can be used to calculate LV mass. Despite its simplicity, the ubiquitous use of the linear dimension method [American Society of Echocardiography (ASE) corrected formula] is limited when compared to the two-dimensional (2D) area length (A-L) method with less reliance on geometrical assumptions (7). It is important to establish whether the use of different estimates of LV mass influence categorisation and derived geometry of the LV in an athlete.

Rugby football league (RFL) is a high intensity sport, utilising both dynamic (50%–75%) and static (10%–20%) components (16). Previous work by our group has characterised the RFL athletes heart using echocardiography by demonstrating a predominance of a normal geometry using the linear method of LV mass derivation and the 2TC classification system (17). In view of this, the aims of this study are two-fold, (1) to compare the linear method and the A-L method for assessment of LV mass in elite rugby players and (2) establish how the differences in methodology impact the classification of LV geometry using both 2TC and 4TC classification systems.

## Methods

### Study population and design

Following ethical approval by the ethics committee of Liverpool John Moores University, male RFL athletes provided written informed consent to participate in the study. Athletes were recruited consecutively and data was collected as part of mandatory pre-participation cardiac evaluation, with participants also completing a medical questionnaire to document any cardiovascular symptoms, family history of SCD or other cardiovascular disease. Participants were required to abstain from exercise training or recreational activity for at least 6 h prior to the investigation and data acquired in a resting state at a single testing session. Screening results were reported by a sports cardiologist with clinical referrals made for any participant requiring further cardiac evaluation.

### Procedures

#### Anthropometry

Anthropometric assessment was undertaken prior to testing. This included height (Seca 217, Hannover, Germany) and body mass measurements (Seca supra 719, Hannover, Germany) with body surface area (BSA) calculated via the Mosteller equation (18).

#### Conventional 2D echocardiography

Conventional 2D echocardiographic images were acquired using a commercially available ultrasound (Vivid Q, GE Medical, Horten, Norway) with a 1.5–4 MHz phased array transducer. Two experienced sonographers (LF/DO) acquired images with participants laying on their left side in the lateral decubitus position, in adherence to ASE guidelines (7). Images were stored as raw digital imaging and communications in medicine (DICOM) format and exported to an offline workstation (Echopac, Version 110.0.2, GE Healthcare, Horten, Norway) for follow up analysis by the same experienced sonographers (LF and DO). Measurements were made in accordance with ASE recommendations (7). A comprehensive assessment of LV wall thickness was employed. Eight measurements were taken at the basal and mid-levels in the parasternal short axis at end diastole (infero-septum, antero-septum, posterior wall and lateral wall). The mean wall thickness (MWT) was calculated from the average of the 8 segments. LV end diastolic volume (LVEDV) was calculated using the Simpson's Biplane summation of discs method from both apical four and two chambers. Concentricity was calculated as (LV mass/LVEDV^0.667^) for application to the 4TC system.

To calculate LV mass, the linear method and the A-L method were both calculated. The linear dimension method was calculated via the ASE corrected formula (19): Left ventricular mass = 0.8 × 1.04 × [(IVS + LVID + PWT)3 − LVID3] + 0.6 g. Left ventricular internal diameter (LVID), interventricular septum (IVS) and posterior wall thickness (PWT) were measured in 2D from the parasternal long-axis view at end-diastole, perpendicular to the LV long axis and measured at the mitral valve leaflet tips (7). The relative wall thickness (RWT) was calculated as (2×PWT)/LVID.

The A-L method was calculated using the equation where the volume of the LV myocardium is calculated from the myocardial area, the average myocardial wall thickness and the LV length. From the parasternal short axis view at the papillary muscle level, the total epicardial area (*A*^1^) and the total endocardial area (*A*^2^) were traced at end diastole, excluding the papillary muscles. The myocardial area (*A*_m_) was obtained from subtracting the total epicardial area (*A*^1^) and LV cavity area (*A*^2^). By assuming a circular area, the average mean wall thickness (*t*) was calculated as $\sqrt{\frac{A^{1}}{\pi }}-b$ where *b* is the SAX radius $\sqrt{\frac{A^{2}}{\pi }}$. From the apical 4-chamber view, the LV long axis length was measured from the mitral annulus to the LV apex at end diastole (*l*). Left ventricular mass was then calculated using the following equation, LV mass = 1.05 {[5/6 *A*_1_ (l + *t*)] − [5/6 *A*_2_ (*l*)]} (7).

### Categorisation

**Figure 1:**
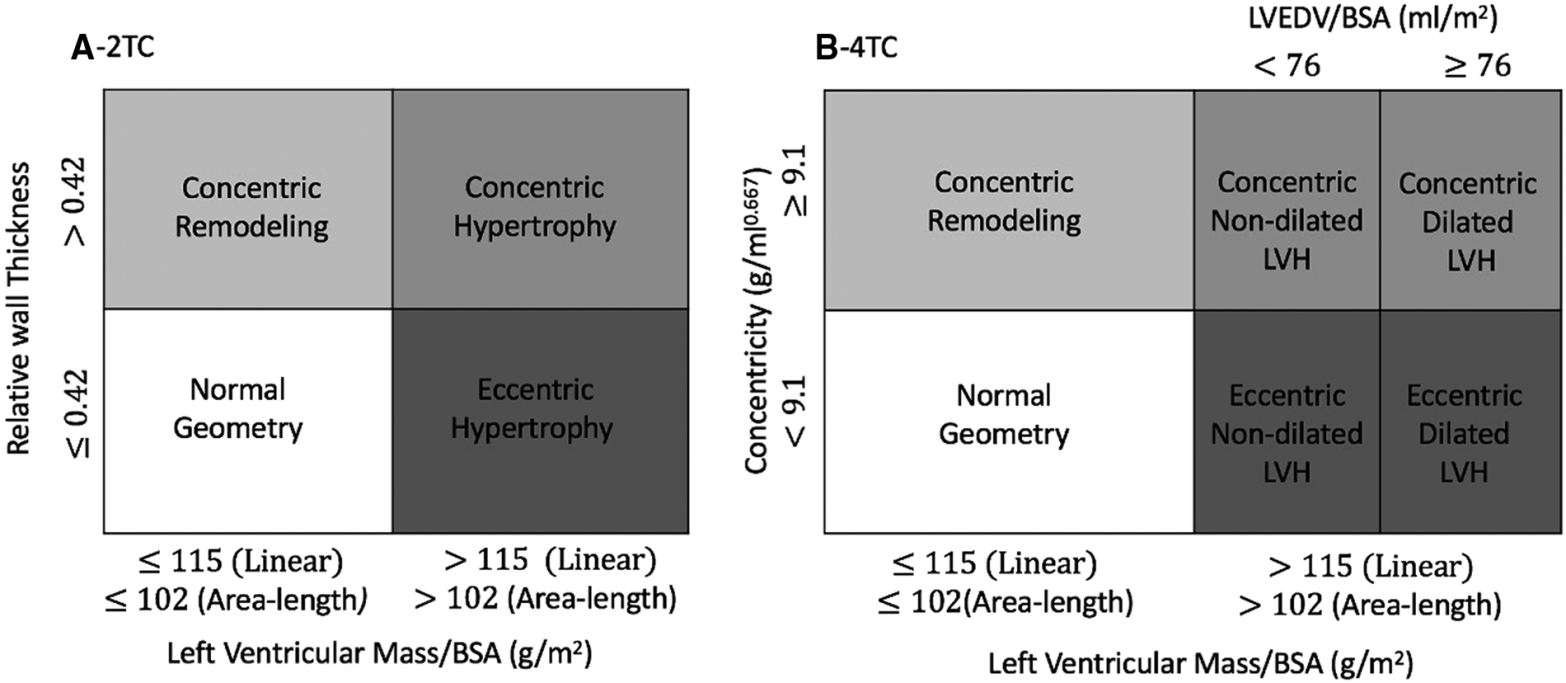
Schematic illustration of A) the two-tiered classification (2TC) and B) four-tiered classification (4TC) of left ventricular hypertrophy based on both the linear method and area-length method. BSA indicates body surface area; LVEDV, left ventricular end-diastolic volume; and LVH, left ventricular hypertrophy.

Left ventricular hypertrophy was defined as LV mass/BSA >115 g/m^2^ for both 2TC and 4TC systems when using the linear dimension method and LV mass/BSA >102 g/m^2^ when using the A-L method (7). Concentric remodelling/concentric hypertrophy were defined as RWT >0.42 in the 2TC. For the 4TC system, echocardiographic thresholds for increased concentricity were ≥9.1 g/ml^2/3^ and dilatation for increased LVEDV index >76 ml/m^2^ (7) (see Figure 1).

### Statistical analysis

Study data were collected and managed using REDCAP electronic data capture tools hosted at Liverpool John Moores University. All echocardiographic data are presented as mean ± SD and ranges. Statistical analyses were performed using a commercially available software package (SPSS, Version 23.0 for Windows, Illinois, USA). A single paired t-test was used to compare the two mass calculations for systemic bias and Bland-Altman agreement was used to establish absolute bias and limits of agreement (LOA). Based on the different classifications the percentage of athletes with specific geometry were presented for 2TC and 4TC as determined from both methods for calculation of LV mass.

## Results

Two hundred and twenty (220) male RFL athletes aged 25 ± 5 (14–34 years) were recruited into the study. All demographic data are presented in Table 1. The absolute and indexed values for LV structure using both the linear and A-L method are presented in Table 2.

**Table 1:** Participant demographics.

Demographics	Mean ± SD
Age (years)	25 ± 5
Height (cm)	181 ± 10
Weight (kg)	90.4 ± 13.2
BSA (m^2^)	2.1 ± 0.2
Training (hours per week)	17 ± 8

**Table 2:** Conventional indices of LV structure.

Parameter	Mean ± SD
Relative wall thickness	0.33 ± 0.04
Mean wall thickness (mm)	8.7 ± 0.8
Mean wall thickness (area equation) (mm)	9.7 ± 1.8
LVEDV (ml)	150 ± 25
LVEDVi (ml/m^2^)	91 ± 10
LVM (ASE equation) (g)	181 ± 34
LVMi (ASE equation) (g/m^2^)	85 ± 13
LVM (area-length equation) (g)	197 ± 34
LVMi (area-length equation) (g/m^2^)	92 ± 13
Concentricity (ASE equation) (g/ml^0.667^)	6.4 ± 1.2
Concentricity (area-length equation (g/ml^0.667^)	7.0 ± 1.1

### Comparison of left ventricular mass

**Figure 2:**
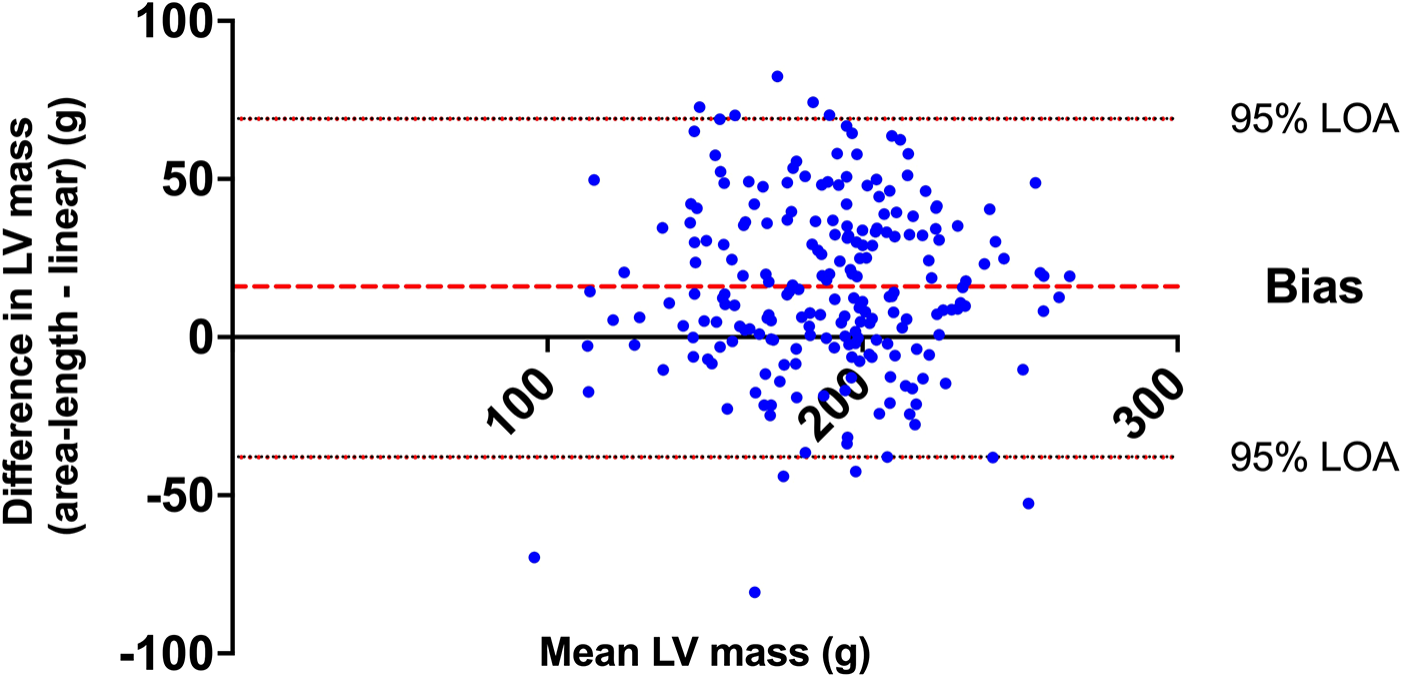
Bland-Altman analysis of LV mass derived from area-length and linear methods.

The linear method provided lower values of absolute (181 ± 34 g vs. 197 ± 34; *p* < 0.0001) and indexed (85 ± 13 g/m^2^ vs. 92 ± 13; *p* < 0.0001) measures of LV mass compared to the A-L method. This difference was reciprocated with lower values of mean wall thickness (8.7 ± 0.8 mm vs. 9.7 ± 1.8; *p* < 0.0001) and concentricity (6.4 ± 1.2 g/ml^0.667^ vs. 7.0 ± 1.1; *p* < 0.001) for linear method compared to A-L method. Bland-Altman analysis of LV mass demonstrated a bias of 16 ± 27 g with 95% LOA of −38 to 69 g (see Figure 2).

### Classifications of LV geometry

**Figure 3:**
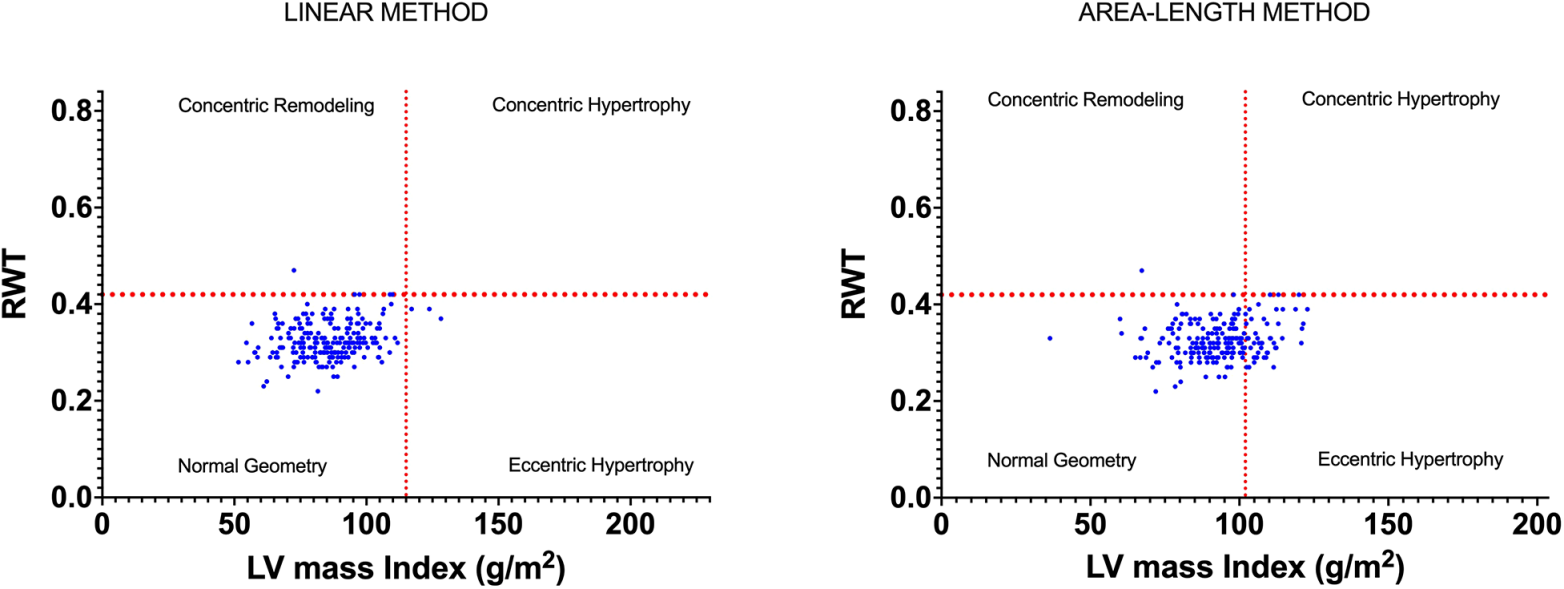
LV geometry classifications based on LVMi and RWT as derived from linear and area-length methods.

Allocation to the 2TC, using the linear method, resulted in 98.2% of participants being classified as having normal geometry, 1.4% as having eccentric hypertrophy, 0.5% as having concentric remodelling and 0% as having concentric hypertrophy. Use of the A-L method altered the prevalence to 80% of participants being categorised as having normal geometry, 19.5% having eccentric hypertrophy, 0.5% having concentric remodelling and 0% having concentric hypertrophy (see Figure 3).

**Figure 4:**
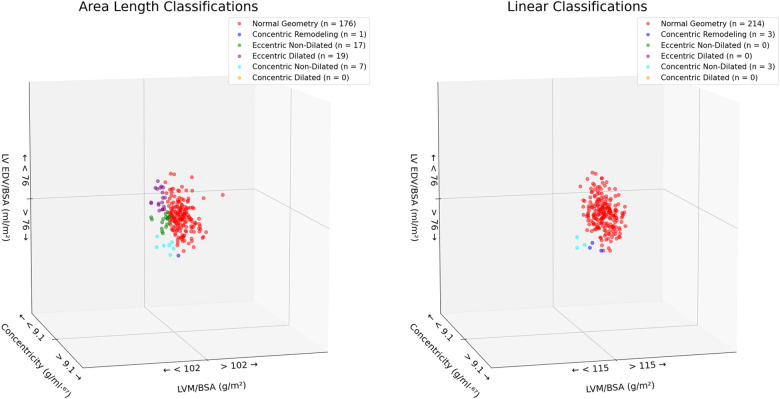
LV geometry classifications based on LVMi and concentricity as derived from linear and area-length methods.

Allocation to the 4TC, using the linear method, resulted in 97% of rugby players being classified as having normal geometry, 1.4% as having concentric remodelling and 1.4% as having concentric non-dilated left ventricular hypertrophy (LVH). Zero percent of participants were classified as having concentric dilated LVH, eccentric non-dilated LVH and eccentric dilated LVH. Use of the 2D Area-length method altered the prevalence to 80% of rugby players being categorised as having normal geometry, 8.6% having eccentric dilated LVH, 7.7% eccentric non-dilated LVH, 3.2% concentric non-dilated LVH, 0.5% concentric remodelling and 0% being categorised as having concentric dilated LVH (see Figure 4 and clips in the Supplementary Data).

## Discussion

This is the first study to compare methods for calculation of LV mass and subsequent derived geometry in a physiological model of the athlete's heart. There was a systematically higher LV mass as determined by the A-L compared to the linear method. When applying both classification systems the linear method categorised approximately 18% more rugby players as having normal geometry compared to the A-L method. Whereas the A-L method categorised more athletes as having eccentric hypertrophy in the 2TC and more eccentric dilated and non-dilated LVH in the 4TC. These findings have implications when considering the application of echocardiography in pre-participation screening or secondary care follow-up of an athlete utilising current guideline recommendations.

### Comparison of LV mass

The measurement of LV mass is integral to the routine echocardiographic assessment of the patient and is advocated by professional guidelines (7, 20). It is used to support the diagnosis of conditions (21), aid management and guide therapy (22) and provide important prognostic data (23). It has also been recommended in the cardiac assessment of the athlete to help differentiate physiological from pathological adaptation at both pre-participation screening and in secondary care follow-up (24). This has been driven by research studies that have aimed to establish normal ranges for LV mass and subsequent geometric classifications in heterogenous athletic populations (2). Of these numerous studies, many of them provide a measurement of LV mass obtained from linear wall thicknesses either using M-mode or 2D. The linear equation uses measurements from a single level of the ventricle and therefore does not fully reflect the distribution of wall thickness from the rest of the LV walls (anterior/inferior, inferoseptum or lateral), from the mid-level or the length of the ventricle. Data from 3D imaging in triathletes have demonstrated a physiologically scaled increase in size throughout the whole of the LV with a maintenance of the overall shape (25) whilst a cMRI study in marathon runners highlights the proportional increase in LV length and normal sphericity (26). Mixed sporting disciplines such as RFL are defined by their relative contribution of isometric and isotonic activity being related to alternate phases of dynamic and static workload and hence these athletes from various field positions provide a unique and heterogeneous combination of cardiac stimuli for adaptation (27). Importantly though the LV mass values obtained from the A-L method here are similar to those observed from absolute 3D echocardiographic derived LV mass data from adult athletes from variable sporting disciplines (28) and may, therefore, be more representative of the athletes heart. The higher values obtained in this study from the A-L method compared to the linear method are in contrast to the literature in non-athletes where studies have demonstrated lower values obtained from the A-L method (29). These data have been adopted by the echocardiographic fraternity/professional bodies and hence the inclusion of absolute lower cut-off values for the A-L method in guideline documents (7, 30, 31). It is important to note however that the specific cut-offs adopted for 2D derived methods of LV mass by the ASE are based on the “truncated ellipsoid method” from only 84 normal participants (29) rather than the A-L method used in this study. The lack of available data in this regard is problematic and highlights the importance of standardisation and consistency but also the potential implications on our understanding of the true nature of physiological athletic adaptation.

The early validation of linear equations for derivation of LV mass was achieved using necropsy findings and specifically M-mode echocardiography in small populations of no LVH (*n* = 34) and pathological LVH (*n* = 18) (19). These data provided the evidence for the currently recommended ASE corrected linear equation. Since that early study, technological advancements in echocardiography have significantly enhanced 2D imaging with superior spatial and temporal resolution whilst overcoming many of the technical limitations of M-mode. In view of this, professional bodies *also* provide support for the use of 2D linear measurements of LV cavity and wall thicknesses (7, 31) and for the subsequent derivation of LV mass using the same linear equation. This lack of standardisation is however problematic as 2D derived cavity values are smaller than those derived from M-mode (32) and there is no reflection of this in the established cut-offs for LV mass (7). This mismatch between methodology and validated ranges may also, in part, explain the systematically lower values obtained from linear derived LV mass in this study alongside the unique balanced nature of physiological LV hypertrophy and the lack of validation in the athlete's heart. The potential implications for this are significant with possible under-reporting of hypertrophy and the ensuing prognostic/diagnostic issues. This issue is exacerbated further due to the integration of RWT in the classification of geometry which suffers from the same issues regarding lack of normal 2D linear derived ranges. Ganau et al. (1992) proposed a cut-off of 0.41 at 95% and 0.44 for 99% percentiles respectively from the distribution of 225 “normal” adult participants using M-mode echocardiography (8). The adoption by professional bodies of a cut-off value of 0.42 has not been further validated particularly using 2D derived linear measurements or in an athletic population and therefore may have reduced specificity in these settings and populations.

### Two-tier vs. four-tier

The integration of LV mass and RWT are used to determine LV geometry using the established 2TC classification system and its application in the young athletic population has allowed us to better understand the nature of physiological cardiac remodelling (3, 33). Previous work by our group in a sub-sample (*n* = 139; age 24 ± 4 years) of the cohort presented in this study demonstrated a predominance for normal geometry using 2TC and linear (2D) derivation of LV mass with only 1.4% and 0.7% of athletes demonstrating eccentric hypertrophy and concentric remodelling respectively and no athletes presenting with concentric hypertrophy (17). In this study we demonstrate similar findings however when using the A-L method for calculation of LV mass and the associated cut-offs there is a significant impact on the number of athletes allocated with eccentric hypertrophy (18%) with no effect on concentric remodelling and hypertrophy compared when using the linear method in the same athletes. The original work of Morganroth et al. (34) demonstrated a dichotomous nature of LV adaptation with concentric hypertrophy in young strength trained athletes and eccentric hypertrophy in young endurance athletes. The concentric limb of this hypothesis has since been refuted based on our developing knowledge of the physiological cardiac stimuli of acute and chronic exposure to exercise training and the mixed nature of sporting disciplines (5, 35) and regardless of the method used to calculate LV mass our findings using 2TC in RFL athletes support this. In contrast, eccentric hypertrophy is an adaptation in response to elevated preload and is associated with well-documented training adaptations of increased blood/plasma volume in endurance sports (36). This concept is also confirmed in our cohort however the evidence presented here suggests that through the potential underestimation of LV mass via linear (2D) measurements, the prevalence of eccentric hypertrophy is likely to be more common in athletes of mixed discipline than previously reported.

The application of the 4TC has been used to better define LV geometry in athletes (12, 13) and utilises concentricity alongside LVEDV. When using the linear (2D) method to determine LV mass, our data demonstrates disparate findings to those obtained via the 2TC. There is a predominance for normal geometry but no athletes with eccentric hypertrophy and conversely 2.8% of athletes with concentric remodelling/hypertrophy. However, when using the A-L method to calculate LV mass, eccentric dilated/non-dilated becomes more prevalent (16.3%) with fewer athletes with concentric remodelling/hypertrophy (0.5%). These are important data and highlight the benefit of both the A-L method combined with the 4TC and further supports our physiological understanding of cardiac adaptation in these athletes. The 4TC also demonstrates the contribution of chamber dilatation to the increase in LV mass and provides further detail on adaptation, i.e., including length and biplane volumes. Previous studies have demonstrated that 33% of elite cyclists were observed to have eccentric (dilated) hypertrophy compared to only 3% of sub-elite cyclists (13) whilst Trachsel et al. (2018) demonstrated that those athletes with greatest training volumes and performance were more likely to have eccentric dilated hypertrophy (12). Our data presents a balanced distribution of eccentric hypertrophy, i.e., with and without dilatation highlighting the heterogeneous presentation of cardiac adaptation in RFL athletes which could be related to position or variable cardio-respiratory fitness. These clear benefits of including the 4TC in the assessment of the athlete's heart are further enhanced by using the A-L method for LV mass derivation and hence removing the reliance of linear measurements.

### Clinical implications

Echocardiography is used routinely in the assessment of the athlete during pre-participation screening where it aids the differentiation of physiological from pathological adaptation. The assessment of LV mass is integral to defining geometry using the 2TC (24) and therefore the data presented here has important implications. The likely underestimation of LV mass values using the linear method with 2D measurements impact on geometry and hypertrophy. It is reassuring that the prevalence of concentric remodelling and concentric hypertrophy is not affected using the A-L method and hence the grey-area to hypertrophic cardiomyopathy is not compounded. There is however an increase in prevalence of eccentric hypertrophy which increases the differential from dilated cardiomyopathy particularly if utilising the 4TC and in the presence of reduced systolic function. We have previously presented an association between strain, ejection fraction and chamber size (17) in RFL athletes with those athletes with bigger cavities having lower EF and strain. It is important to be aware of this association and consider a low threshold for exercise testing to demonstrate functional reserve in athletes with eccentric LVH and reduced systolic function (37). Going forward it is imperative that recommendations highlight the differences and limitations of current echocardiographic methods for deriving LV mass and the role of more comprehensive classifications systems for defining geometry whilst maintaining a sensible approach to standardisation and interpretation of these data.

Athletes that are referred to secondary care for follow-up due to inconclusive echocardiography or suspicion of cardiac disease are often subjected to cardiac magnetic resonance imaging (cMRI) (38). cMRI is well established as the gold-standard for determining LV mass however values have been shown to be lower than those obtained from echocardiography (27) and has been confirmed in a meta-analysis of athletes of mixed sporting discipline (39). The data presented here provides additional “food for thought” regarding multi-modality assessment of the athlete's heart and the lack of interchangeability between cMRI and echocardiography. It is essential that clinicians are aware of these differences particularly when defining normality and attempting to differentiate from pathological adaptation.

### Limitations

There are some limitations in this study. We targeted male, young RFL athletes only and although there are variabilities in body size and field positions the external validity of our findings are limited. That aside RFL athletes are representative of the unique mixed demands of team sport and therefore provide important insight in this wider demographic. Further studies should aim to focus on reproducing and expanding these findings in other athlete demographics including athletes of black ethnicity where concentric LVH has been reported to be more prevalent.

## Conclusion

The linear (2D) and A-L method for calculation of LV mass in RFL athletes are not interchangeable; with systematically higher values obtained using the A-L method. The application of these methods significantly impacts geometry classification with more athletes presenting with eccentric hypertrophy using 2TC and eccentric dilated/non-dilated using 4TC. This data also provides greater insight into the magnitude and nature of adaptation of the RFL athlete's heart. Further studies should be aimed at establishing the association of A-L methods of LV mass and application of the 4TC to the multi-factorial demographics of the athlete.

## Data Availability

The original contributions presented in the study are included in the article/**Supplementary Material**, further inquiries can be directed to the corresponding author.
