# Supplementary material for: Comparison of echocardiographic methods for calculating left ventricular mass in elite rugby football league athletes and the impact on chamber geometry
Source: Front Sports Act Living. 2023 Sep 13;5:1270444. doi: 10.3389/fspor.2023.1270444 (PMC10533919; doi:10.3389/fspor.2023.1270444)
Supplement: Supplementary file 1 [file Table1.docx]

**Supplementals**

Clip Area-Length classification (.gif)

<https://ljmu-my.sharepoint.com/personal/spsdoxbo_ljmu_ac_uk/_layouts/15/onedrive.aspx?ga=1&id=%2Fpersonal%2Fspsdoxbo%5Fljmu%5Fac%5Fuk%2FDocuments%2FArea%20Length%20Classifications%2Egif&parent=%2Fpersonal%2Fspsdoxbo%5Fljmu%5Fac%5Fuk%2FDocuments>

Clip Linear classification (.gif)

<https://ljmu-my.sharepoint.com/personal/spsdoxbo_ljmu_ac_uk/_layouts/15/onedrive.aspx?ga=1&id=%2Fpersonal%2Fspsdoxbo%5Fljmu%5Fac%5Fuk%2FDocuments%2FLinear%20Classifications%2Egif&parent=%2Fpersonal%2Fspsdoxbo%5Fljmu%5Fac%5Fuk%2FDocuments>
